# Genomic and functional impact of Trp53 inactivation in JAK2V617F myeloproliferative neoplasms

**DOI:** 10.1038/s41408-023-00969-6

**Published:** 2024-01-04

**Authors:** Panhong Gou, Duanya Liu, Saravanan Ganesan, Evelyne Lauret, Nabih Maslah, Veronique Parietti, Wenchao Zhang, Véronique Meignin, Jean-Jacques Kiladjian, Bruno Cassinat, Stephane Giraudier

**Affiliations:** 1 Inserm UMR-S 1131, Hôpital Saint-Louis, Paris, France; 2 Université de Paris Cité, Paris, France; 3 Université de Paris, Institut Cochin, Inserm U1016, CNRS UMR 8104, Paris, France; 4 Service de Biologie Cellulaire, Hôpital Saint-Louis, Assistance Publique–Hôpitaux de Paris, Paris, France; 5 INSERM/CNRS US53/UAR2030, Institut de Recherche Saint-Louis, Paris, France; 6 Histo-pathological Department, Hôpital Saint-Louis, Paris, France; 7 Centre Investigations Cliniques, Hôpital Saint-Louis, Paris, France

**Keywords:** Haematopoietic stem cells, Cell signalling

## Abstract

Classical myeloproliferative neoplasms (MPNs) are characterized by the proliferation of myeloid cells and the risk of transformation into myelofibrosis or acute myeloid leukemia (AML) and *TP53* mutations in MPN patients are linked to AML. However, JAK2V617F has been reported to impact the *TP53* response to DNA damage, suggesting potential overlapping role of TP53 inactivation in MPN. We established a mouse model showing that JAK2V617F/Vav-Cre/Trp53^−/−^ mice displayed a similar phenotype to JAK2V617F/Vav-Cre mice, but their proliferation was outcompeted in competitive grafts. RNA-Seq revealed that half of the genes affected by JAK2V617F were affected by p53-inactivation, including the interferon pathway. To validate this finding, mice were repopulated with a mixture of wild-type and JAK2V617F (or JAK2V617F/Vav-Cre/Trp53^−/−^) cells and treated with pegylated interferonα. JAK2V617F-reconstituted mice entered complete hematological remission, while JAK2V617F/Vav-Cre /Trp53^−/−^-reconstituted mice did not, confirming that *p53* loss induced interferon-α resistance. KEGG and Gene Ontology analyses of common deregulated genes showed that these genes were mainly implicated in cytokine response, proliferation, and leukemia evolution, illustrating that in this mouse model, the development of MPN is not affected by TP53 inactivation. Taken together, our results show that many genetic modifications induced by JAK2V617F are influenced by TP53, the MPN phenotype may not be. *Trp53* loss alone is insufficient to induce rapid leukemic transformation in steady-state hematopoiesis in JAK2V617F MPN, and *Trp53* loss may contribute to interferon resistance in MPN.

## Introduction

Classical myeloproliferative neoplasms (MPNs) are hematopoietic disorders characterized by clonal proliferation of mature myeloid elements, which manifest clinically as excess red blood cells, platelets, and/or white blood cells (WBCs) [1]. Mutations in kinases such as Janus kinase 2 (JAK2) have been identified in the majority of patients with MPNs, underscoring the importance of activated transduction signaling in the pathogenesis of these disorders [2–6]. A role of cellular tumor antigen p53 (p53) in JAK2V617F signaling–induced cell proliferation has been postulated. JAK2 strongly inhibits the stabilization of p53 after induction of DNA damage through the increase in E3 ubiquitin-protein ligase Mdm2 (MDM2) translation, which is dependent on the phosphatidylinositol 3-kinase (PI3K)/AKT/mTOR pathway in cell lines and primary cells from MPN patients [7]. Moreover, JAK2V617F-dependent cell growth is inhibited by MDM2 inhibitors, thus suggesting an important role of the MDM2/p53 axis in JAK2V617F-mediated proliferation [8, 9].

However, inactivation and mutations in *TP53* have also been associated with post-MPN acute myeloid leukemia (AML) transformation (in 25%-50% of cases) [10–13], suggesting that inactivation of the *TP53* tumor suppressor could be a cooperating contributing event in combination with signaling due to JAK2 gain-of-function mutation during the transformation process.

Preclinical data from *TP53*-inactivated mouse models overexpressing JAK2V617F suggest that the combination of this sole abnormality in a JAK2V617F-overexpressing cell could be sufficient to cause acute transformation in 1.5 to 4 months [14, 15], but during the MPN phase development, the MPN phenotype of JAK2V617F/Trp53^−/−^ mice does not seem to be highly different from the phenotype of JAK2V617F-only mice. This finding was confirmed recently by Li et al. using a transgenic mouse model [16]. However, in this model, when JAK2V617F expression is only driven by its promoter, transformation occurs after a latency of 135 days. This latency suggests that p53 inactivation could be more involved in genetic instability and a long-term transformation process than in short-term leukemic transformation. Moreover, clinical data tempered the “2-hits only” hypothesis because AML is uncommon (∼5% of associated malignancies) in patients with Li-Fraumeni syndrome associated with germline *TP53* mutations [17, 18]. Additionally, patients with chronic-phase MPN harboring *TP53* mutations who have a long survival time do not have an increased risk of transformation [19–21].

Recent studies suggest that the quantity of *TP53*-mutated cells could be responsible for the transformation, more so than the existence of *TP53* mutation; small *TP53*-mutated clones (less than 10% of Variant allele frequency) could have a neutral impact while larger clones could be detrimental [19–22].

To better define the role of *TP53* in the JAK2V617F phenotype and the impact of *TP53* inactivation in MPN evolution and the response to treatment to reduce the clonal size, we studied mice with *Trp53* inactivation and JAK2V617F endogenous mutation presenting a full phenotype of MPN (i.e., thrombocytosis, polycythemia, and leukocytosis).

Here, our data suggest that (1) p53 may be un-responsible for the MPN progressive phenotype, despite a large set of gene expression deregulations being affected by *Trp53* inactivation; (2) *Trp53* inactivation facilitates clonal progression; and (3) *Trp53* inactivation induces resistance to interferon-α (IFN-α) therapy in MPN but is not sufficient to induce direct transformation of chronic-phase MPN, leading us to hypothesize that acute transformation of such pathologies requires more than two oncogenic hits.

## Methods

### Mouse model

Mice were bred and maintained in pathogen-free conditions in the animal facilities unit at the Université de Paris Cité. All procedures performed has been carried out in accordance with national (n° 2013-118) and European (n°2010/63/UE) directives for animal experiments. Animals were handled according to the guidelines of institutional animal care committees using protocols approved by the “Comité d’Ethique Experimentation Animal Paris-Nord” (no. 121) (project number #24499-2020030501008440). Animals were housed at our animal facility (UMS Saint-Louis US53/UAR2030, Institut de Recherche Saint-Louis, Paris, France) in accordance with animal welfare and ethical guidelines (accreditation number B75-10-08).

The floxed JAK2V617F mice were kindly provided by J.L. Villeval [23]. We crossed floxed JAK2V617F mice with the Vav-Cre mice [24–26] to induce the JAK2 mutation in hematopoietic cells. We also crossed the floxed JAK2V617F mice with Trp53^−/−^ mice kindly provided by Dr. Kawakita [27] to get JAK2^floxed/+^/Trp53^+/^^−^ mice. In parallel, Vav-Cre mice were crossed with Trp53^−/−^ mice and backcrossed in Trp53^−/−^ mice to obtain Vav-Cre/Trp53^−/−^ mice. JAK2^floxed/+^/Trp53^+/^^−^ mice were then crossed with Vav-Cre/Trp53^−/−^ mice to obtain Cre-induced recombination and finally JAK2V617F expression at the JAK2 locus and under the JAK2 promoter in hematopoietic cells harboring Trp53 inactivation.

### BM and spleen analysis

Mice were euthanized by cervical dislocation. Bones (femurs and tibias) and spleens were harvested, and muscle and tendon tissue were removed using a scalpel and Kimwipes. BM and spleen samples were used for cell count, cytometry, and histology.

BM fraction was flushed out using a syringe containing 1× PBS with 2% fetal bovine serum (FBS). The resulting cell suspension was filtered through a 40-µm cell strainer (Corning, NY, USA) and pelleted by centrifugation. For mechanical grinding, spleens were smashed and ground between rough sides of frosted glass slides, and cells were collected in DMEM containing 2% FBS. Cell suspensions were passed through a 40-µm cell strainer, and cells were re-suspended in DMEM containing 10% FBS. Suspensions were subjected to cytometry analysis after red blood cell lysis.

For bone marrow (BM) transplant, hind limbs were extracted and cleaned. Total BM was flushed and then passed through a 40-µm cell strainer to obtain a single-cell suspension. A mix of cells from the different mice was prepared in a 1:1 (CD45.2 JAK2V617F-VavCre cells and CD45.1 wild-type (WT) cells or CD45.2 JAK2V617F/Vav-Cre/Trp53^−/−^ and WT cells) or 2:1:1, 3:2:1, or 8:7:1 (CD45.1 WT cells, CD45.2 JAK2V617F/Vav-Cre cells, and CD45.2 JAK2V617F/Vav-Cre/Trp53^−/−^) ratio. A mixture of 5×10^6^ cells was resuspended in a total volume of 0.2 mL and transplanted into a minimum of 5 irradiated (9 Gy) CD45.1/CD45.2 C57/BL6 recipient mice. After reconstitution, chimerism analysis was performed using cytometry and colony genotyping to distinguish between CD45.2 JAK2V617F/Vav-Cre cells and CD45.2 JAK2V617F/Vav-Cre/Trp53^−/−^ colonies.

Murine pegylated-IFN-α (PharmaEssentia Corp, Taiwan) was subcutaneously injected every week at doses of 600 ng in 0.1 mL phosphate buffered-saline (PBS)/mouse. Alternatively, 100 µL of 10 mg/mL bromodeoxyuridine (BrdU) were intraperitoneally injected (BD Pharmingen, USA). Orbital plexus blood from anesthetized mice was collected in ethylenediaminetetraacetic acid tubes. Blood cell counts were determined using an automated blood counter (MS9, Schloessing Melet, Cergy-Pontoise, France). Blood samples were used for cytometry analysis. BM cells were collected by flushing both femurs and tibias. Spleens were weighed, and single-cell suspensions were prepared.

### Cytometry analysis

Erythrocytes were lysed using BD FACS Lysing Solution (BD Biosciences, Franklin Lakes, NJ, USA) before flow cytometry staining. Total BM and spleen cells were stained with Zombie UV Fixable Viability Kit, (BioLegend, Le Pont de Claix Cedex, France) for 20 min at room temperature in the dark and thereafter stained with biotinylated anti-mouse Lineage Cocktail antibodies for 30 min (BioLegend). After washing, cells were stained for 30 min using the following monoclonal antibodies in the BD Horizon Brilliant staining buffer: BV 510 anti-mouse Ly-6A/E (Sca-1-), D7, (BioLegend); BB700 rat anti-mouse CD117 (PerCP), 2B8, (BD Biosciences); BV711 anti-mouse CD48, HM48-1; PE/Cy7 anti-mouse CD150 (SLAM), TC15-12 F12.2 (BioLegend); Alexa Fluor 647 rat anti-mouse CD34, RAM34 (RUO) (BD Biosciences); and BV786 rat anti-mouse CD16/CD32, 2.4G2 (RUO) (BD Biosciences).

For proliferation analysis, surface staining was performed as described above, and cells were processed for cell proliferation according to the manufacturer’s recommendations (BD Pharmingen BrdU Flow kits) with Alexa Fluor 488 anti-BrdU Antibody, 3D4 (BioLegend). Data analysis was performed at the Cochin Cytometry and Immunobiology Facility Cytometry analyses on a Fortessa LSRII cytometer (BD Biosciences), and analyses were done on Kaluza software.

### Genomic analysis

RNA was extracted from cells sorted into LT-HSC (Lin-sca+c-kit+CD150-CD48-), ST-HSC (Lin^−^sca^+^c-kit^+^CD150^+^CD48^−^), MPP (Lin^−^sca^+^c-kit^+^CD150-CD48^+^), CMP (Lin^+^sca^+^c-kit^+^CD34^+^CD16/32^−^), MEP (Lin^+^sca^+^c-kit^+^CD34^−^CD16/32^−^) and GMP (Lin^+^sca^+^c-kit^+^CD34^+^CD16/32^+^) populations from WT mice, JAK2V617F/Vav-Cre transgenic mice, and JAK2V617F/Vav-Cre/Trp53^−/−^ transgenic mice using a BD FACSAria III cell sorter (BD Biosciences) and a QIAcube device (Qiagen). RNA was quantified using Nanodrop (100 ng/uL) and qualified in a Bioanalyzer (Agilent). The RNA-Seq analysis was performed at the Cochin Institute genomic platform (Paris).

After the paired end (2 × 75 bp) sequencing, a primary analysis based on AOZAN software (ENS, Paris) was applied to demultiplex and to control the quality of the raw data (based on FastQC modules / version 0.11.5). The fastq files were then aligned using the STAR algorithm (version 2.7.1a) on the GRCm38 reference from Ensembl (release 101) and quality control of the alignment realized with Picard tools (version 2.8.1). STAR parameters were the following: --sjdbOverhang 74 --twopassMode Basic --outFilterType BySJout --quantMode TranscriptomeSAM. Reads were then counted using RSEM (v1.3.1) with the AlignedtoTranscriptome bam files, and the statistical analyses on the read counts were performed with the DESeq2 package version 1.24.0 to determine the proportion of differentially expressed genes between 2 conditions. During the statistical analysis, we filtered out annotations where there were less than 3 samples with normalized counts greater than or equal to 10.

Pre-ranked lists of gene fold changes were generated in DESeq2 between 3 groups. Analyses were pursued with fast gene set enrichment analysis (FGSEA) implemented in the R Bioconductor package applied to the ranked gene lists. Principal component analysis (plotPCA), volcano plots (EnhancedVolcano), and gesaplots were all generated in R.

### Statistical analysis

Results are presented as mean ± SD. Data were analyzed through two-tailed Student *t*-test using Graph Pad 8.0 software. In all figures *=*p* < 0.05, **=*p* < 0.01, ***=*p* < 0.001.

## Results

### Constitutive expression of JAK2V617F/Vav-Cre in Trp53-inactivated cells results in MPN-like phenotype

The JAK2V617F conditional allele mice were crossed with Vav-Cre transgenic mice to generate JAK2V617F/Vav-Cre mice. To assess the function of *Trp53* inactivation in the background of JAK2V617F, the JAK2V617F/Vav-Cre/Trp53^−/−^ mice were bred as described in the mouse model section.

**Fig. 1:**
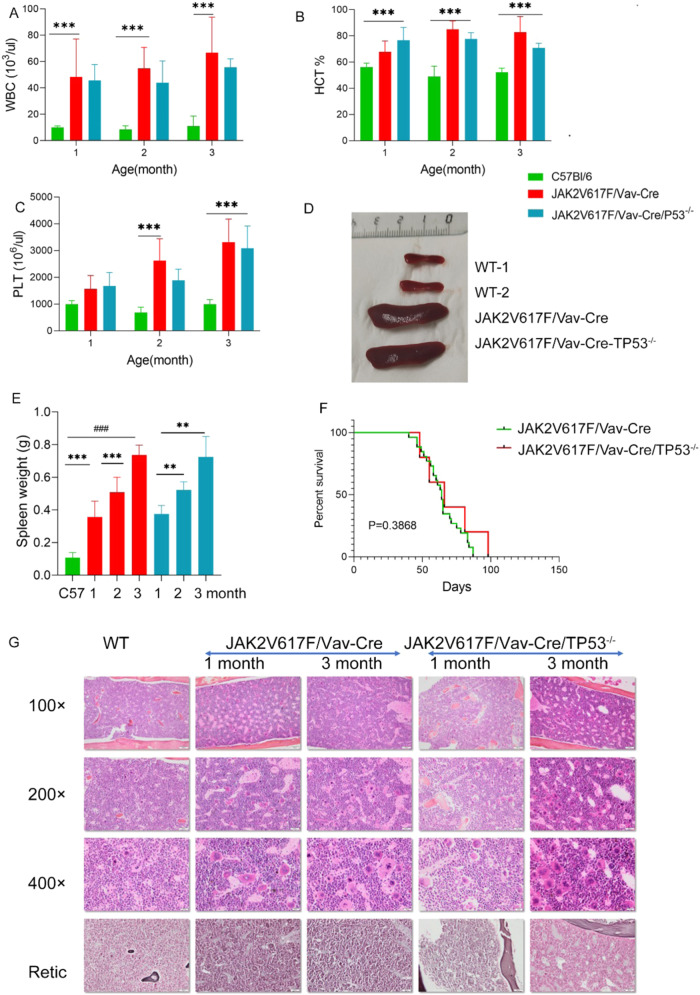
The inactivation of *Trp53* does not modify the JAK2V617F-induced MPN phenotype. **A**–**C** Data of cell count of peripheral blood (PB) from C57Bl/6, JAK2V617F/Vav-Cre, and JAK2V617F/Vav-Cre/Trp53^−/−^ mice, graph represents pooled mice from three independent experiment, each with 3-4 mice per group. (**A**: white blood cell, **B**: hematocrit, **C**: platelet). **D** The spleen of C57Bl/6, JAK2V617F/Vav-Cre, and JAK2V617F/Vav-Cre /Trp53^−/−^ mice. The graph represents from three independent experiments, each with 3-4 mice per group. **E** The spleen weight from C57Bl/6, JAK2V617F/Vav-Cre, and JAK2V617F/Vav-Cre /Trp53^−/−^ mice at 1 to 3 months old. Graph represents pooled mice from three independent experiment, each with 3-4 mice per genotype. **F** Percent survival of JAK2V617F/Vav-Cre and JAK2V617F/Vav-Cre /Trp53^−/−^ mice. **G** Histopathologic characterization of the bone marrow (BM). The first column is the BM of C57Bl/6 mice, the second column is the BM from 1-month-old JAK2V617F/Vav-Cre mice, the third column is BM from 3-month-old JAK2V617F mice, the fourth column is BM from 1-month-old JAK2V617F/Trp53^−/−^ mice, and the fifth column is BM from 3-month-old JAK2V617F/Trp53^−/−^ mice. The first row to third row respectively the 100×, 200×, and 400× objectives. The samples on the fourth row were silver stained, and the samples on the other rows were hematoxylin and eosin stained. Images were obtained using a microscope with an Olympus camera.

Blood counts demonstrated a similar phenotype in JAK2V617F/Vav-Cre mice and JAK2V617F/Vav-Cre/Trp53^−/−^ mice. WBC counts for JAK2V617F/Vav-Cre and JAK2V617F/Vav-Cre/Trp53^−/−^ mice (43.12 ± 12.10 G/L and 48.30 ± 12.83 G/L, respectively) were significantly higher (4-fold) than those of control mice (9.94 ± 1.18 G/L) 1 month after birth and continued to rise during the second (42.57 ± 16.59 G/L, p = 0.001 and 54.78 ± 15.91 G/L, p = 0.0004 for JAK2V617F/Vav-Cre and JAK2V617F/Vav-Cre/Trp53^−/−^ mice, respectively) and third months (52.12 ± 6.46 and 66.73 ± 27.07 G/L for JAK2V617F/Vav-Cre and JAK2V617F/Vav-Cre/Trp53^−/−^ mice, respectively) (Fig. 1A). Hematocrit in JAK2V617F/Vav-Cre/Trp53^−/−^ and JAK2V617F/Vav-Cre mice was higher (61.89% ± 9.70% and 67.87% ± 8.19%, respectively) at 1 month after birth compared to 56.12% ± 3.07% in control mice (p = 0.006 and p = 0.001, respectively) (Fig. 1B). Platelet counts were also higher than normal at 1-month in JAK2V617F/Vav-Cre and JAK2V617F/Vav-Cre/Trp53^−/−^ mice. Platelet count for JAK2V617F/Vav-Cre mice continued to rise from 1571 ± 491 G/L to 3497 ± 948 G/L, and JAK2V617F/Vav-Cre/Trp53^−/−^ mice displayed the same trend of increasing platelet count from 1 to 3 months (Fig. 1C). Spleen weights were enlarged in the same order of magnitude (5-fold at 3 months) in both JAK2V617F/Vav-Cre and JAK2V617F/Vav-Cre/Trp53^−/−^ mice and increased gradually during the time in both backgrounds (Fig. 1D, E) compared to control mice (*p* = 0.6519 × 10^−8^ and *p* = 0.324 × 10^−4^ at 3 months, respectively).

BM and spleen histological analyses were performed at 1 and 3 months. BM histological analysis, as previously reported [23], illustrated that the megakaryocytic density of JAK2V617F/Vav-Cre mice was higher than that of normal mice (Fig. 1G). No difference was noticed between JAK2V617F/Vav-Cre/Trp53^−/−^ and JAK2V617F/Vav-Cre mice. Because mice were relatively young, reticulin fiber deposits were not prominent in JAK2V617F/Vav-Cre or JAK2V617F/Vav-Cre/Trp53^−/−^ mice at 1 or 3 months (Fig. 1G).

Histological spleen analysis revealed that JAK2V617F/Vav-Cre mice lost their classical histology: removal of white pulp, significant expansion of red pulp, and increased number of megakaryocytes. Increased number and size of clusters of immature red lineage precursors were noticed in the 2 types of mice (data not shown).

No evidence of blast infiltration was noticed at 1 and 3 months old in blood, BM, or spleen samples in both JAK2V617F mice and JAK2V617F/Vav-Cre/Trp53^−/−^ mice (Fig. S1).

Lastly, survival analysis showed that the median survival time for JAK2V617F/Vav-Cre mice was around 2 months when no treatment (or venesection) was performed; *Trp53* inactivation did not shorten JAK2V617F/Vav-Cre mice’s (already short) lifespan (Fig. 1F).

Collectively, these data show that heterozygous endogenous JAK2V617F expression in hematopoietic cells leads to hyperplasia of mature and maturing erythroid, granulocytic, and megakaryocytic cells in blood and hematopoietic tissues, an MPN phenotype that may not be influenced by the P53 genetic inactivation.

### *Trp53* inactivation confers a proliferative advantage to JAK2V617F/Vav-Cre cells

**Fig. 2:**
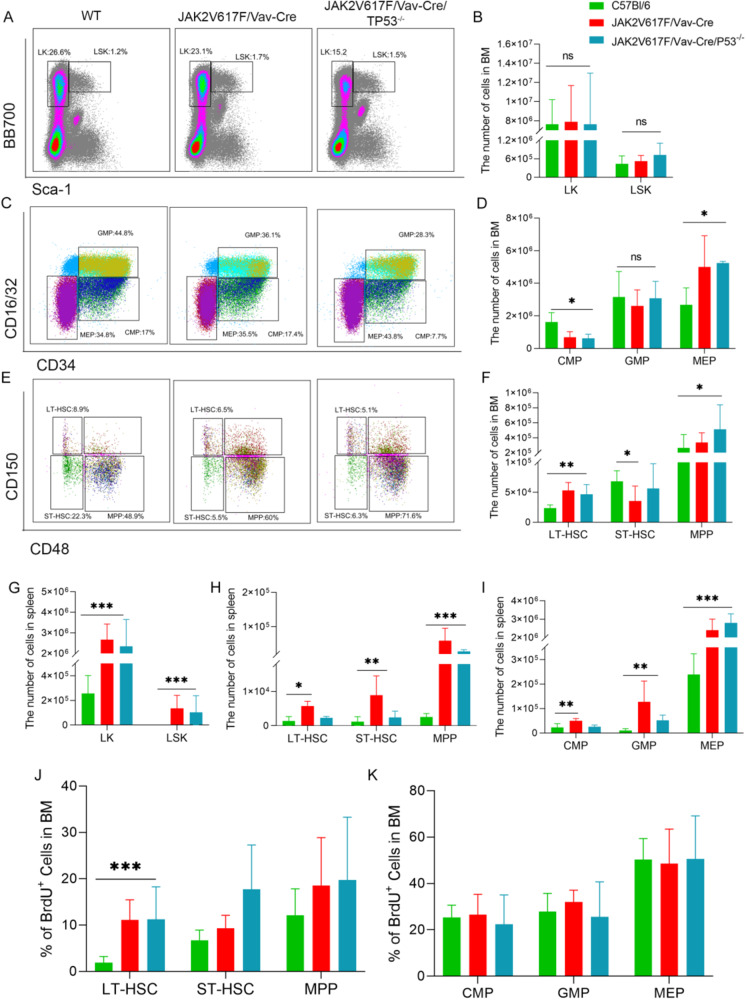
Inactivation of *Trp53* does not lead to expansion of immature progenitors in JAK2V617F mice. **A** Representative flow cytometric plots of LK (Lin^−^sca^−^c-kit^+^) and LSK (Lin^−^sca^+^c-kit^+^) cell percentages from the bone marrow (BM) of C57B/l, JAK2V617F/Vav-Cre, and JAK2V617F/Vav-Cre /Trp53^−/−^ mice. **B** The total number of LK and LSK cells from the BM of C57B/l, JAK2V617F/Vav-Cre, and JAK2V617F/Vav-Cre /Trp53^−/−^ mice. Graph represents pooled mice from three independent experiments, each with 3-4 mice per genotype. **C** Representative flow cytometric plots of CMP (Lin^+^sca^+^c-kit^+^CD34^+^CD16/32), GMP (Lin^+^sca^+^c-kit^+^CD34^+^CD16/32^+^), and MEP (Lin^+^sca^+^c-kit^+^CD34^−^CD16/32^−^) cell percentages from the BM from C57B/l, JAK2V617F/Vav-Cre, and JAK2V617F/Vav-Cre/Trp53^−/−^ mice. **D** Total numbers of LT-HSC, ST-HSC, and MPP cells from the BM of C57B/l, JAK2V617F/Vav-Cre, and JAK2V617F/Vav-Cre /Trp53^−/−^ mice. Graph represents three independent experiments, each with 3-4 mice per genotype. **E** Representative flow cytometric plots of LT-HSC (Lin^−^sca^+^c-kit^+^CD150^−^CD48^−^), ST-HSC (Lin^−^sca^+^c-kit^+^CD150^+^CD48^−^), and MPP (Lin^−^sca^+^c-kit^+^CD150^−^CD48^+^) cell percentages from the BM of C57B/l, JAK2V617F/Vav-Cre, and JAK2V617F/Vav-Cre /Trp53^−/−^ mice. **F** Total numbers of CMP, GMP, and MEP cells from the BM of C57B/l, JAK2V617F/Vav-Cre, and JAK2V617F/Vav-Cre /Trp53^−/−^ mice. Graph represents pooled mice from three independent experiment, each with 3-4 mice per group. **G** The total number of LK and LSK cells from the spleen of C57B/l6, JAK2V617F, and JAK2V617F/p53^−/−^ mice. **H** The total number of CMP, GMP, and MEP cells from the spleen of C57B/l6, JAK2V617F/Vav-Cre, and JAK2V617F/Vav-Cre /Trp53^−/−^ mice. Graph represents pooled mice from three independent experiment, each with 3-4 mice per genotype. **I** The total number of LT-HSC, ST-HSC, and MPP cells from the spleen of C57B/l6, JAK2V617F/Vav-Cre, and JAK2V617F/Vav-Cre /Trp53^−/−^ mice. Graph represents pooled mice from three independent experiment, each with 3-4 mice per genotype. **J** The percentage of BrdU^+^ cells of LT-HSC, ST-HSC, and MPP from the BM of C57B/l6, JAK2V617F/Vav-Cre, and JAK2V617F/Vav-Cre /Trp53^−/−^ mice. Graph represents pooled mice from three independent experiment, each with 3-4 mice per genotype. **K** The percentage of BrdU^+^ cells of CMP, GMP, and MEP from the BM of C57B/l6, JAK2V617F/Vav-Cre, and JAK2V617F/Vav-Cre /Trp53^−/−^ mice. Graph represents pooled mice from three independent experiment, each with 3-4 mice per genotype. Data are mean ± SD *p < 0.05, **p < 0.01, ***p < 0.001.

As previously described [23], endogenous JAK2V617F expression increases early stages of differentiation (cell numbers) and proliferation. Immature cell populations frequencies (LT-HSC, ST-HSC, MPP, CMP, MEP, and GMP cells) and absolute numbers in JAK2V617F/Vav-Cre and JAK2V617F/Vav-Cre/Trp53^−/−^ mice, as well as their proliferative capacities in vivo, were assessed by flow cytometry at 2 to 3 months of age (Fig. 2, Tables S1–3, and Fig. S2). At these ages, BM cellularity was not affected by either molecular change. Mice demonstrated no significant changes in the numbers of Lin^−^ c-Kit^+^ (LK), Lin^−^Sca-1^+^c-Kit^+^ (LSK), or GMP (Fig. 2B, F) but had an increase in most immature LT-HSC, MPP, and MEP cells in the BM compared to normal mice (Table S1 and Fig. 2B, D, F; *p* = 0.16, *p* = 0.012, *p* = 0.02, and *p* = 0.031, respectively). These increases in cell numbers were observed in both JAK2V617F/Vav-Cre mice and JAK2V617F/Vav-Cre/Trp53^−/−^ mice, without any significant statistical differences between the two groups. In the spleen, the number of LK, LSK, CMP, GMP, and MEP cells and the more immature LT-HSC, ST-HSC, and MPP cells were drastically increased as described above. These features were also found in JAK2V617F/Vav-Cre/Trp53^−/−^ mouse spleens (Fig. 2G–I and Table S2; LK, *p* = 0.0002; LSK, *p* = 0.012; CMP, *p* = 0.003; GMP, *p* = 0.008; MEP, *p* = 3.34 × 10^−^^6^; LT-HSC, *p* = 0.04; ST-HSC, *p* = 0.008; and MPP, *p* = 0.0003). These results show that *Trp53* inactivation does not modify the amplification of all stages of differentiation observed in endogenous JAK2V617F/Vav-Cre cells, suggesting that JAK2V617F/Vav-Cre immature cell proliferation, just like the MPN phenotype, is not changed by *Trp53*-inactivation.

To confirm the lack of proliferative advantage induced by *Trp53* inactivation in a JAK2V617F/Vav-Cre background in hematopoietic stem and progenitor compartments, BrdU analysis was conducted. The BrdU-positive S-phase fraction of DNA-synthesizing cells was statistically increased only in BM LT-HSC from JAK2V617F/Vav-Cre mice, regardless of *Trp53* status (Fig. 2J) (C57BL6: 1.87% ± 1.36%; JAK2V617F/Vav-Cre: 11.10% ± 4.36%, *p* = 0.0068; JAK2V617F/Vav-Cre/Trp53^−/−^: 15.15% ± 2.33%, *p* = 0.044) as previously described for the JAK2V617F mice [23]. The percentage of BrdU cells in other populations (ST-HSC, MPP, CMP, GMP, and MEP) did not differ significantly in WT, JAK2V617F/Vav-Cre, and JAK2V617F/Vav-Cre/Trp53^−/−^ mice (Fig. 2K).

**Fig. 3:**
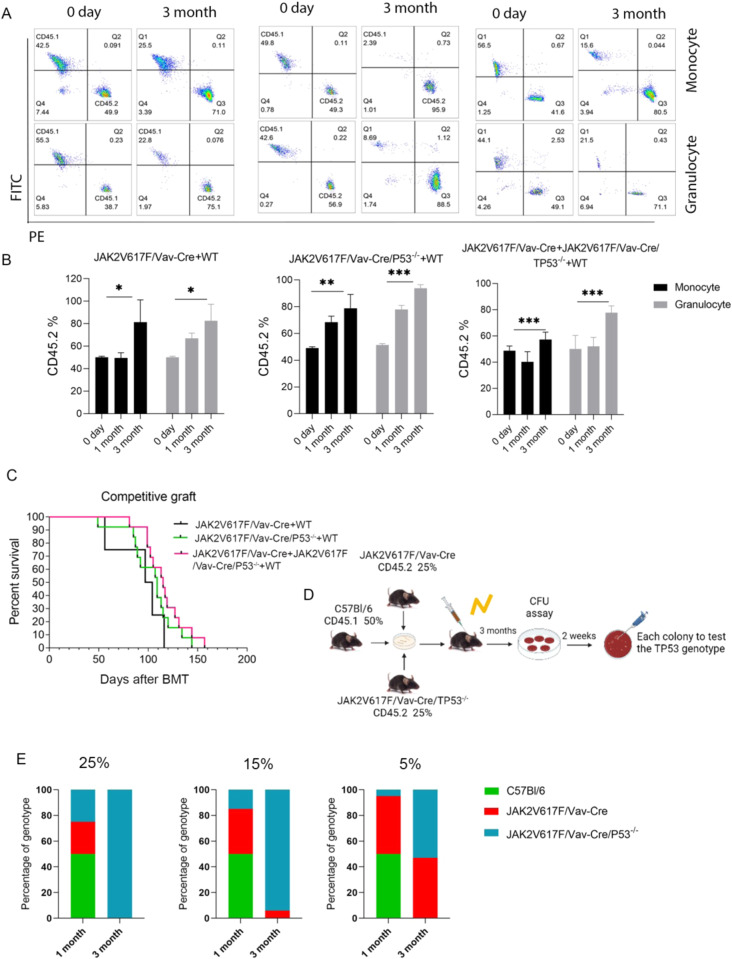
Competitive graft of JAK2V617/Vav-Cre F and JAK2V617F/Vav-Cre /Trp53^−/−^ murine cells. **A** Representative flow cytometric plots of CD45.1 and CD45.2 at different time points (day 0 and month 3) of JAK2V617F/Vav-Cre (50%) + wild type (WT) (50%) competitive graft (left), JAK2V617F/Vav-Cre /Trp53^−/−^ (50%) + WT (50%) competitive graft (middle), and JAK2V617F/Vav-Cre (25%) + JAK2V617F/Vav-Cre /Trp53^−/−^ (25%) + WT (50%) competitive graft from at least two independent experiments. **B** The percentage of CD45.2 in competitive graft groups, each with 7-10 mice per genotype. **C** The survival of different competitive graft groups. **D** The schematic of triple competitive graft. **E** The percentage of genotyping in different competitive grafts from at least two independent experiments. The group of 25% JAK2V617F/Vav-Cre (25%) + JAK2V617F/Vav-Cre /Trp53^−/−^ (25%) + WT(50%) (left); the group of 15% JAK2V617F/Vav-Cre (35%) + JAK2V617F/Vav-Cre /Trp53^−/−^ (15%) + WT(50%) (middle); the group of 5% JAK2V617F/Vav-Cre (45%) + JAK2V617F/Vav-Cre /Trp53^−/−^ (5%) + WT (50%) (right). Data are mean ± SD *p < 0.05, **p < 0.01, ***p < 0.001.

Finally, to functionally compare hematopoietic reconstitution capacities of JAK2V617F/Vav-Cre and JAK2V617F/Vav-Cre/Trp53^−/−^ cells, double and triple competitive repopulations were performed using CD45.2 JAK2V617F/Vav-Cre mice, CD45.2 *Trp53*-inactivated JAK2V617F/Vav-Cre mice as donors and normal CD45.1 BM cells in CD45.1+ CD45.2 lethally irradiated mice. Regardless of *Trp53* status, the JAK2V617F cells exhibited a huge advantage over WT cells; blood chimerism analysis performed 3 months after graft showed >80% of JAK2V617F/Vav-Cre CD45.2 cells (JAK2V617F/Vav-Cre + CD45.1 competitive graft, 50% ± 1% [day 0] to 81% ± 19.8% [3 month] in monocytes [p = 0.047] and 50% ± 1.5% [day 0] to 82.6% ± 14.6% [3 month] in granulocytes [p = 0.025]; Fig. 3A left and 3B left). JAK2V617F/Vav-Cre/Trp53^−/−^ + CD45.1 competitive graft showed the same increase in the JAK2V617F chimerism: 49% ± 1% (day 0) to 78.9% ± 10.3% (3 month) in monocytes (p = 0.003) and 51.3% ± 1.2% (day 0) to 93.9% ± 2.5% (3 month) in granulocytes (p = 1.92×10^−^^7^; Fig. 3A middle, Fig. 3B middle). At least, triple competition with JAK2V617F/Vav-Cre CD45.2 cells (25%) + JAK2V617F/Vav-Cre/Trp53^−/−^ CD45.2 cells (25%) + WT CD45.1 cells (50%) were performed Chimerism of JAK2V617F was as followed: 48.8% ± 3.5% (day 0) to 57% ± 10.2% (3 month) in monocytes (p = 0.027) and 50.3% ± 5.6% (day 0) to 77.7% ± 5.3% (3 month) in granulocytes (p = 0.002 l Fig. 3A right and 3B right). In all cases, mice developed an MPN phenotype during the first 2 months and half-life expectancy of engrafted mice was reduced to 100 to 120 days (Fig. 3C).

The BM chimerism 3 months after transplant of normal BM cells (50%) together with different mixes of JAK2V617F/Vav-Cre/Trp53^−/−^ and JAK2V617F/Vav-Cre cells were examined by flow cytometry. Previous experiments (by us and other researchers) have shown that a graft with 50% of WT BM cells and 50% of JAK2V617F/Vav-Cre induces a quick MPN disorder and chimerism of 80%-100% of JAK2V617F/Vav-Cre cells 3 months after reconstitution, illustrating the JAK2V617F-induced proliferative advantage/invasion property of these pathological cells [23]. In all cases, chimerism at 3 months highlighted the growth advantage of JAK2V617F/Vav-Cre cells, regardless of the ratio between the JAK2V617F/Vav-Cre and JAK2V617F/Vav-Cre/Trp53^−/−^ mutated grafts. We took advantage of this property and transplanted irradiated mice with either a mixture of 50% WT cells + 25% JAK2V617F/Vav-Cre cells + 25% JAK2V617F/Vav-Cre/Trp53^−/−^ cells (ratio 2:1:1; Fig. 3D), a mixture of 50% WT cells + 35% JAK2V617F/Vav-Cre cells + 15% JAK2V617F/Vav-Cre/Trp53^−/−^ (ratio 3:2:1), or a mixture of 50% WT cells + 45% JAK2V617F/Vav-Cre cells + 5% JAK2V617F/Vav-Cre/Trp53^−/−^ (ratio 10:9:1). These ratios led us to inject approximately 262, 157, and 52 JAK2V617F/Vav-Cre//Trp53^−/−^ LT-HSC cells in the recipients. In all cases (but when mice received only 10 LT-HSCs, (data not shown)), these cell numbers were sufficient to generate MPN with complete penetrance. Thereafter, BM cells from 3-month-old transplanted animals were seeded in methylcellulose for 10-12 days, and myeloid colonies were picked and genotyped for *JAK2* and *Trp53* status. All BM colonies harbored the JAK2V617F/Vav-Cre recombination confirming previous data that transplanting at least 30 LT-HSCs is necessary to fully develop an MPN phenotype in a mouse model and respectively 100% (Fig. 3E left), 94% (Fig. 3E middle), and 47% (Fig. 3E right) were inactivated for *Trp53*, illustrating the competitive advantage of JAK2V617F/Vav-Cre/Trp53^−/−^ over JAK2V617F cells. Moreover, these triple competitive BM transplants demonstrate in vivo that 52 JAK2V617F/Vav-Cre/Trp53^−/−^ cells can overgrow 472 JAK2V617F/Vav-Cre/ LT-HSC, suggesting an over 9-fold capacity to invade the hematopoietic system over JAK2V617F-only cells when JAK2V617F LT-HSCs have approximatively the same advantage over WT cells as reported previously [23]. Thus, JAK2V617F/Vav-Cre/Trp53^−/−^ cells could have a 20-fold proliferative advantage over WT cells during stress hematopoiesis (i.e., after BM transplant).

These results confirmed that JAK2V617F provides a competitive advantage to hematopoietic cells at the early stages of differentiation and, interestingly, highlighted that *Trp53* inactivation associated with JAK2V617F mutation enabled an added competitive advantage to JAK2V617F mutation alone during reconstitution (stress hematopoiesis) that was not evident in BrdU experiments.

### Genomic analysis reveals *Trp53*-dependent and -independent deregulations induced by JAK2V617F as observed in vivo

**Fig. 4:**
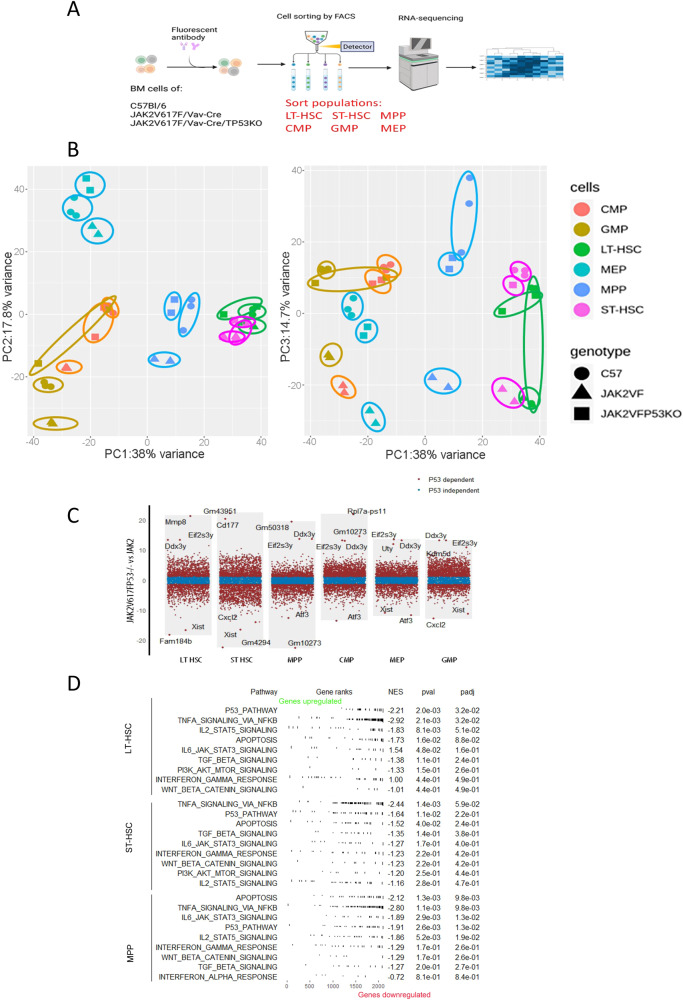
*Trp53*-related and -unrelated JAK2V617F/Vav-Cre deregulations in vivo. **A** Schematic representation of RNA-seq analysis. **B** Principal Component Analysis with different populations from C57B/l6 (WT), JAK2V617F/Vav-Cre and JAK2V617F/Vav-Cre /Trp53^−/−^ mice: Green dots: LT-HSC, violet dots: ST-HSC, blue dots: MPP, Light-green dots: MEP, orange dots: CMP, brown dots: GMP. Triangles: JAK2V617F/Vav-Cre cells, Rounds: WT cells, Squares: JAK2V617F/Vav-Cre /Trp53^−/−^ cells). **C** The volcano diagram identifies the *p53*-related and -unrelated genes from LT-HSC, ST-HSC, and MPP cell populations. **D** Nine most important p53 dependent pathways in JAK2V617F stem cell compartments using GSEA analysis (LT-HSC, ST-HSC, and MPP).

To better understand changes in immature cells related to *Trp53* inactivation in a JAK2V617F context, RNA-Seq analysis was performed on steady state mice after immature populations (LT-HSC, ST-HSC, MPP, CMP, MEP, GMP) from normal, JAK2V617F/Vav-Cre, and JAK2V617F/Vav-Cre/Trp53^−/−^ mice (Fig. 4A) were cell sorted.

Unsupervised analysis classified the type of cell compartment (Fig. S3A). The principal component analysis confirmed the unsupervised findings (Fig. 4B). RNA-Seq distinguished each type of immature compartment with a gradient in principal component (PC)1, PC2, and PC3 (38%, 17.8%, and 14.7% variance, respectively). We identified statistically significant difference genes between JAK2V617F and WT, termed JAK2V617F-specific genes (|Log FC JAK2V617F vs WT | 1.2). On the basis of the JAK2V617F-specific gene, we identified p53-related JAK2V617F-specific genes (LogFC JAK2V617F/Trp53^−/−^ vs JAK2V617F > 1.2), and p53-unrelated JAK2V617F-specific genes (LogFC JAK2V617F/Trp53^−/−^ vs JAK2V617F ≤ 1.2) between JAK2V617F and JAK2V617F/Trp53^−/−^ compartments (Fig. 4C). On JAK2V617F-specific genes, more than half of them were p53-related (LT:2133/3496, ST:2713/3496, CMP:2829/4276, GMP:2588/3549, and MEP:2116/4229, Table S4), which is consistence with the study about the main role of p53 in JAK2 signaling [7].

We took advantage of these data to analyze *p53*-related or -unrelated JAK2V617F-deregulated pathways. For *p53*-associated genes, a gene set enrichment analysis (GSEA) with hallmark gene sets from the molecular signature database was performed (Fig. 4D, Fig. S3B). It showed that apoptosis, TNF/NFκB signaling, and the p53 pathway were downregulated in JAK2V617FT/Trp53^−/−^ mice when compared with JAK2V617F mice confirming previous results illustrating the role of inflammation in TP53 inactivated cells evolution [28]. This is consistent with the finding that JAK2V617F/Trp53^−/−^ have higher competitive engraftment. Trp53 knockout is associated with the downregulation of genes in inflammation/immune function-related pathways, including interferon response, which may be why some MPN patients harboring with P53 mutation were non-responsive to IFN treatment.

For *p53*-unrelated genes, we performed a GSEA analysis with ontology gene sets (M5) from the molecular signature database MsigDB (Fig. S4A). While there were few p53-unrelated genes (Table S4), there was an enrichment for cell cycle-related pathways (Fig. S4B).

### IFN-α therapy of JAK2V617F/Vav-Cre/Trp53^−/−^ MPN in vivo

**Fig. 5:**
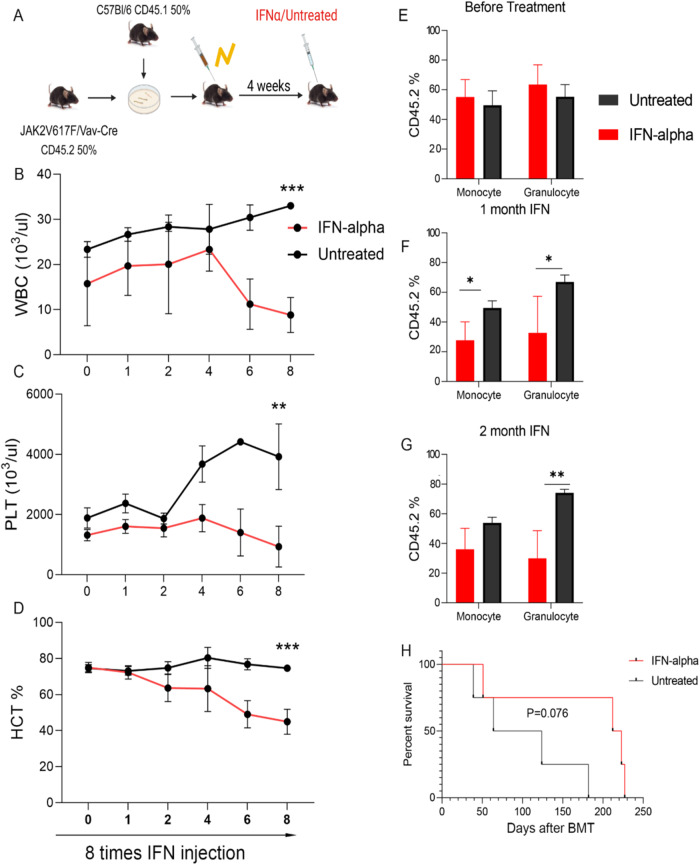
JAK2V617F/Vav-Cre mice respond to the interferon-α (IFN-α) treatment. **A** The schematic of the experiment. **B**–**D** The number of peripheral blood (PB) between IFN-α treatment and untreated mice in JAK2V617F/Vav-Cre donor mice and CD5.1 donor mice. **B** The number of white blood cells (WBC). **C** The number of platelets (PLT). **D** The percentage of hematocrit (HCT). Graph represents at least two independent experiments of each time point, each with 4 to 5 mice per group. **E**–**G** The percentage of CD45.2 (JAK2V617F/Vav-Cre cells) in blood chimerism of treated and untreated mice. Graph represents at least two independent experiments of each time point, each with 4 to 5 mice per group. **E** before treatment, **F** after 1 month of IFN treatment, and **G** after 2 months of IFN treatment. **H** The survival of treated and untreated mice. Data are mean ± SD *p < 0.05, **p < 0.01, ***p < 0.001.

**Fig. 6:**
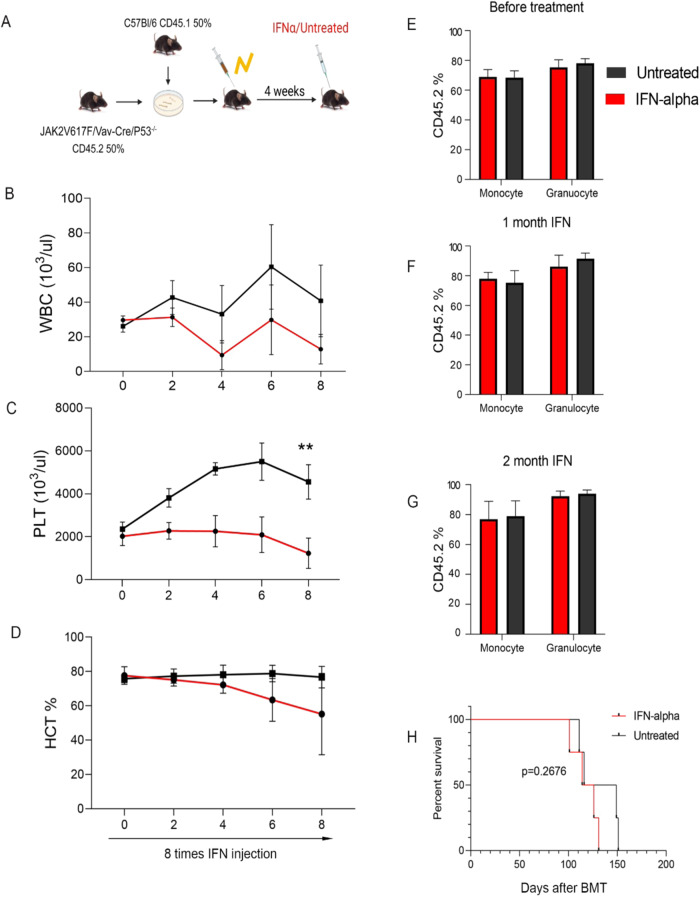
JAK2V617F/Vav-Cre Trp53^−/−^ mice do not respond to interferon-α (IFN-α) treatment. **A** The schematic of the experiment. **B**–**D** The number of peripheral blood (PB) between IFN-α-treated and untreated mice in JAK2V617F/Vav-Cre/Trp53^−/−^ donor mice and CD5.1 donor mice. **B** The number of white blood cells (WBC). **C** The number of platelets (PLT). **D** The percentage of hematocrit (HCT). Graph represents at least two independent experiments of each time point, each with 4 to 5 mice per group. **E**–**G** The percentage of CD45.2 (JAK2V617F/Vav-Cre/Trp53^−/−^ cell) in blood chimerism of treated and untreated mice. Graph represents at least two independent experiments of each time point, each with 4 to 5 mice per group. **E** before treatment, **F** after 1 month of IFN treatment, and **G** after 2 months of IFN treatment. **H** The survival of treated and untreated mice. Data are mean ± SD *p < 0.05, **p < 0.01, ***p < 0.00.

To further explore the potential role of IFN signaling activation in *p53*-dependent JAK2V617F treatment response, pegylated-IFN-α treatment was initiated in 2 groups of CD45.1 WT recipient mice 4 weeks after competitive transplantation of CD45.1 BM WT cells with 50% JAK2V617F/Vav-Cre (Fig. 5A) or 50% JAK2V617F/Vav-Cre/Trp53^−/−^ CD45.2 BM cells (Fig. 6A). One month after transplantation, when the MPN phenotype was observed, weekly murine pegylated-IFN-α therapy was started and repeated for 8 weeks (once per week). In JAK2V617F/Vav-Cre–transplanted mice, IFN-α treatment induced suppression of leukocytosis (*p* = 0.0023) and normalization of platelet count (*p* = 0.00238) and hematocrit (*p* = 0.0041) after 8 weeks of treatment (Fig. 5B-D), as previously reported [23, 29]. Treatment in the JAK2V617F/Vav-Cre /Trp53^−/−^–transplanted mice also induced a hematological response (Fig. 6B-D). Chimerism analysis confirmed a drastic reduction in the JAK2V617F/Vav-Cre proportion of myeloid cells in the blood of the JAK2V617F/Vav-Cre recipients, (*p* = 0.01; Fig. 5G), but no change was detected in the chimerism analysis despite IFN therapy in the JAK2V617F/Vav-Cre /Trp53^−/−^–recipient mice (*p* = 0.79; Fig. 6G). These hematological and chimerism findings were confirmed by the survival analysis with a slight (albeit not significant, *p* = 0.076) increase in survival for IFN-α-treated JAK2V617F/Vav-Cre mice (Fig. 5I) and not for the IFN-α-treated JAK2V617F/Vav-Cre /Trp53^−/−^–recipient mice (*p* = 0.2676; Fig. 6H).

These results confirm previous reports on the efficacy of IFN-α to hamper JAK2V617F/Vav-Cre cell proliferation with normalization of most hematological parameters and reduce the proliferative advantage of JAK2V617F/Vav-Cre over WT cells, but *Trp53* inactivation abrogates this selective effect of IFN-α of JAK2V617F, in line with the RNA-Seq analysis.

## Discussion

At the biochemical level, cross-talk between JAK2V617F mutation and p53 has been largely described. JAK2V617F reduces p53 levels by inhibiting NPM1 [30]. Direct interactions between p53 and STAT5 can also be inhibited by JAK2V617F [31]. JAK2V617F has also been shown to induce the accumulation of La, increasing its translation and then inhibiting p53 [7]. This JAK2/MDM2 pathway has been clinically reinforced since the MDM2 [9] inhibitor (Nutlin-3) significantly reduces MPN-CD34^+^ cell proliferation when combined with IFN-α [9]. Lastly, other JAK2-dependant transduction pathways such as the RAS/MAPK or AKT/mTOR pathways also regulate p53 functions because ERK phosphorylation upregulates MDM2 expression. Taken together, these results suggest that JAK2V617F mutation and inactivation of p53 could be overlapping events in MPN, suggesting that p53 inactivation should not induce large modifications in chronic MPN phenotypes.

The fact that JAK2 directly regulates and inhibits TP53 function on the one hand but that TP53 inhibition could induce a transformation of JAK2V617F cells on the other hand prompted us to develop a more physiological model of JAK2V617F expression in the context of p53 inactivation in immature BM cells. Our analyses of the biological process and more particularly the MPN phenotype (high leukocytes, red blood cells, and platelets; spleen enlargement, BM histology, and survival) demonstrate the lack of differences between JAK2V617F/Vav-Cre and JAK2V617F/Vav-Cre/Trp53^−/−^ mice, at least during the first months (our severe model of MPN induces death of mice in 3 months and we can not exclude that a prolonged survival would have made possible to highlight an increased risk of transformation in JAK2V617F/Vav-Cre/Trp53^−/−^ mice). This finding directly indicates that the JAK2V617F-induced MPN phenotype is not affected by *p53*-inactivation. In this context, phenotypic modifications due to p53 inactivation in a JAK2V617F context were very mild, reinforcing the hypothesis of overlapping between JAK2V617F activation and p53 inactivation. However, in patients, *TP53* mutations/inactivations are late events, and we cannot definitively rule out that the order of emergence of each mutation could impact the emergence of MPN transformation. Clonal interference could play a role in such transformations.

However, some genetic and phenotypic traits seem to remain p53-related in a JAK2V617F context. First, BM competition between JAK2V617F/Vav-Cre and JAK2V617F/Vav-Cre/Trp53^−/−^ BM cells demonstrate a huge advantage to p53-inactivated cells. It has been suggested previously that 30 LT-HSC are warranted to develop in 100% of mice a JAK2V617F-induced MPN phenotype [23]. Here, we tested this hypothesis. In our study, WT mice harbored 11,027 ± 742 LT-HSCs when JAK2V617F/Vav-Cre or JAK2V617F/Vav-Cre/Trp53^−/−^ mice harbored 33,250 ± 25,000 LT-HSCs, meaning that JAK2V617F mice have approximatively 3 times more LT-HSCs than normal mice. Triple competitive BM transplants with different ratios of WT, JAK2V617F/Vav-Cre, and JAK2V617F/Vav-Cre/Trp53^−/−^ cells, demonstrated a huge proliferative advantage of JAK2V617F/Vav-Cre/Trp53^−/−^ over JAK2V617F/Vav-Cre suggesting that JAK2V617F-induced over-proliferation is p53-dependent. In favor of this hypothesis, genes involved in apoptosis, STAT5, NF-κB, or TGFβ—all pathways known to play a role in stem cell proliferation—are deregulated in JAK2V617F/Trp53^−/−^ stem cells compared to JAK2V617F cells.

Second, a molecular analysis performed during a steady state tends to demonstrate that 44% (from 7% to 77% according to the studied hematopoietic subpopulations) of genes that are deregulated by JAK2V617F are affected by TP53 inactivation because immature populations of JAK2V617F/Vav-Cre/Trp53^−/−^ cells are closer to normal compared to JAK2V617F/Vav-Cre cells. Such a set of genes include the IFN pathway–associated genes that are clearly upregulated in the JAK2V617F context (explaining the high sensitivity of these cells to IFN therapy) and comparatively downregulated when p53 is inactivated, suggesting a lower sensitivity of these cells to IFN therapy. IFN-α treatment increases *TP53* phosphorylation in cells from patients with MPN but also increases phosphorylation of STAT1, p21, PUMA, and Bak proteins, which are known to be involved in apoptotic processes [32, 33]. IFN-α is also known to induce apoptosis of hematopoietic stem cells by the p38 MAPK pathway activation through a p53-dependent mechanism [33]. This finding is consistent with our RNA-Seq analysis and in vivo data that suggest that loss of p53 in the JAK2V617F context causes reduces sensitivity of stem cells to IFN-α treatment. MDM2 inhibitor RG7112 combined with IFN-α significantly decreases the number of JAK2V617F hematopoietic colonies through the activity of the p53 pathway [8] in the mouse model, which may confirm the differential sensitivity to IFN-α therapy in JAK2V617F/Vav-Cre cells compared to WT or JAK2V617F/Vav-Cre/Trp53^−/−^ cells in vivo. This finding suggests that patients harboring clones with p53 mutations in chronic MPN could be less sensitive to an IFN-based regimen, at least when *TP53* is inactivated. This finding will need to be confirmed in IFN-α-treated patients in order to decipher whether screening p53 mutations/inactivation should be performed before IFN-α therapy in humans, just like hypothesized by our group for the use of MDM2 modulators [34, 35].

In conclusion, our results illustrate that a large number of gene expression deregulations in the JAK2V617F context are affected by *p53*-inactivation, but the MPN phenotype is not. This work also illustrates that P53 inactivation in the hematopoietic cells endogenous JAK2V617F context is not sufficient to directly induce the transformation of MPN despite inducing a proliferative advantage over *p53* WT cells in the short-term, but we can not definitively exclude such an effect in the long-term. Our data also suggest that *Trp53* inactivation could induce resistance to IFN therapy, a finding that needs to be confirmed in clinical studies.

## Data policy details

The datasets generated during and analysed during the current study are not publicly available due to new analysis for other purposes in our lab but the raw data are available from the corresponding author on reasonable request.

### Supplementary information

Dataset1

Genomic and functional impact of Trp53 inactivation in JAK2V617F myeloproliferative neoplasms.
