# Supplementary material for: Genomic and functional impact of Trp53 inactivation in JAK2V617F myeloproliferative neoplasms
Source: Blood Cancer J. 2024 Jan 4;14(1):1. doi: 10.1038/s41408-023-00969-6 (PMC10766605; doi:10.1038/s41408-023-00969-6)
Supplement: Supplementary file 1 — Dataset1 [file 41408_2023_969_MOESM1_ESM.rtf]

REAGENT or RESOURCE	SOURCE	IDENTIFIER	
Antibodies			
Zombie UV Fixable Viabiility Kit	Biolegend	Cat:423107	
anti-mouse Lineage Cocktail	Biolegend	Cat:133307	
anti-mouse Ly-6A/E (Sca-1) BV510	Biolegend	Cat:108129	
anti-mouse CD117 BB700	BD Biosciences	Cat:566414	
anti-mouse CD48 BV711	Biolegend	Cat: 103439	
anti-mouse CD150 (SLAM) PE/Cy7	Biolegend	Cat: 115914	
anti-mouse CD34 AF647	BD Biosciences	Cat: 560230	
anti-mouse CD16/CD32 BV786	BD Biosciences	Cat: 740851	
anti-BrdU AF488	Biolegend	Cat: 364106	
anti-Mouse CD45.1 FITC	Biolegend	Cat:110705	
anti-Mouse CD45.2 PE	Biolegend	Cat:109808	
BD Horizon™ Brilliant Stain Buffer	BD Biosciences	Cat: 566349	
APC/Cy7 Streptavidin 	Biolegend	Cat:405208	
Rainbow Calibration Particles(8-Peaks)	BD Biosciences	Cat:559123	
Chemicals, peptides			
BrdU	BD Biosciences	Cat: 566349	
BD Pharm Lyse™ Lysing Buffer	BD Biosciences	Cat: 555899	
BD Perm/Wash Buffer                   	BD Biosciences                 	Cat: 554723           	
			
Experimental models:strains			
C57BL/6(B6)	Jackson Laboratories	Strain:000664	
B6.SJL-Ptprca Pepcb/BoyJ(B6 CD45.1)	Jackson Laboratories	Strain:002014	
B6.Cg-Tg(VAV1-cre)1Graf/MdfJ	Jackson Laboratories	Strain: 035670	
B6.JAK2floxed/+	Hasan et al.	PMID: 23863895	
B6.Trp53-/-	Donehower et al.	PMID: 1552940	
Key resources table


Study design

The number of mice per experiment was determined based on the power calculation of published data. Littermate controls were used when possible. Control and experimental mice were age- and sex-matched. In experiments, all of the data generated were included in the analysis. All experimental findings were replicated, and the number of replicates is indicated in the figure legends.
When performing animal experiments, we minimize bias through appropriate blinding procedures. For feasible blind experiments (for example, the experimental operator is not aware of the genotype of the animal during data collection such as peripheral blood count and flow cytometry staining stage, so as to ensure impartial data collection and analysis) , however, in some cases ,blinding cannot be achieved due to the experimental design or the nature of the measurements involved (For example different groups of mice treated with IFN). 
