# Supplementary material for: Genomic and functional impact of Trp53 inactivation in JAK2V617F myeloproliferative neoplasms
Source: Blood Cancer J. 2024 Jan 4;14(1):1. doi: 10.1038/s41408-023-00969-6 (PMC10766605; doi:10.1038/s41408-023-00969-6)
Supplement: Supplementary file 2 — Genomic and functional impact of Trp53 inactivation in JAK2V617F myeloproliferative neoplasms. [file 41408_2023_969_MOESM2_ESM.docx]

**Genomic and functional impact of Trp53 inactivation in JAK2V617F myeloproliferative neoplasms.**

Short Title: JAK2V617F & Trp53 mutation in MPN

Panhong Gou^1,2^, Duanya Liu^1,2^, Saravanan Ganesan^1^, Evelyne Lauret^3^, Nabih Maslah^1,2,4^, Veronique Parietti^2,5^, Wenchao Zhang^2^, Véronique Meignin^2,6^, Jean-Jacques Kiladjian^1,2,7^, Bruno Cassinat^1,4^, and Stephane Giraudier^1,2,4^

**Supplementary tables**

Table 1. The absolute cell number of each cell population in the bone marrow from wild type, JAK2V617F/Vav-Cre, and JAK2V617F/Vav-Cre/Trp53^-/-^ mice

|  | LSK  (10^5^/mouse) | LT-HSC (10^4^/mouse) | ST-HSC  (10^4^/mouse) | MPP  (10^5^/mouse) | LK  (10^6^/mouse) | CMP  (10^6^/mouse) | MEP  (10^6^/mouse) | GMP  (10^6^/mouse) |
| --- | --- | --- | --- | --- | --- | --- | --- | --- |
| C57Bl/6 | 4.74±2.7 | 2.17±0.61 | 7.52±2.34 | 2.03±0. 98 | 7.64±2.56 | 1.62±0.57 | 3.00±0.86 | 2.64±0.99 |
| JAK2V617F/Vav-Cre | 6.14±2.17 | 4.86±1.84** | 4.19±2.74* | 3.82±1.68 | 8.94±4.45 | 1.07±0.54 | 4.25±1.92 | 2.61±0.98 |
| JAK2V617F/VavCre/  Trp53^-/-^ | 8.35±3.76 | 4.08±1.78* | 5.63±4.14 | 6.24±2.96* | 9.47±4.74 | 0.62±0.25* | 5.24±0.99* | 3.08±1.03 |

LSK, Lin^-^sca^+^c-kit^+^; LT-HSC, Lin^-^sca^+^c-kit^+^CD150^-^CD48^-^; ST-HSC, Lin^-^sca^+^c-kit^+^CD150^+^CD48^-^; MPP, Lin^-^sca^+^c-kit^+^CD150^-^CD48^+^; CMP, Lin^+^sca^+^c-kit^+^CD34^+^CD16/32^-^; MEP, Lin^+^sca^+^c-kit^+^CD34^-^CD16/32^-^; and GMP, Lin^+^sca^+^c-kit^+^CD34^+^CD16/32^+^. Data are mean±SD *p<0.05, **p<0.01, ***p<0.00

Table 2. The absolute cell number of each cell population in spleens from wild type, JAK2V617F/Vav-Cre, and JAK2V617F/Vav-Cre/Trp53^-/-^ mice.

|  | LSK  (10^4^/mouse) | LT-HSC  (10^3^/mouse) | ST-HSC  (10^3^/mouse) | MPP  (10^4^/mouse) | LK  (10^5^/mouse) | CMP  (10^4^/mouse) | MEP  (10^5^/mouse) | GMP  (10^4^/mouse) |
| --- | --- | --- | --- | --- | --- | --- | --- | --- |
| C57Bl/6 | 0.56±1.3 | 1.38±1.2 | 1.35±1.4 | 0.26±0.09 | 3.38±0.96 | 2.16±1.39 | 2.28±0.81 | 0.52±0.18 |
| JAK2V617F/Vav-Cre | 6.30±4.03* | 4.73±2.56* | 4.98±2.72** | 5.00±2.05*** | 27.32±6.66*** | 5.55±1.42** | 24.46±5.37*** | 9.69±5.68** |
| JAK2V617F/VavCre/Trp53^-/-^ | 3.63±0.61*** | 2.33±0.37 | 2.45±1.8 | 2.75±0.54*** | 28.77±5.17*** | 2.57±0.67 | 25.86±5.08*** | 8.14±3.1** |

LSK, Lin^-^sca^+^c-kit^+^; LT-HSC, Lin^-^sca^+^c-kit^+^CD150^-^CD48^-^; ST-HSC, Lin^-^sca^+^c-kit^+^CD150^+^CD48^-^; MPP, Lin^-^sca^+^c-kit^+^CD150^-^CD48^+^; LSK, Lin^-^sca^-^c-kit^+^; CMP, Lin^+^sca^+^c-kit^+^CD34^+^CD16/32^-^; MEP, Lin^+^sca^+^c-kit^+^CD34^-^CD16/32^-^; and GMP, Lin^+^sca^+^c-kit^+^CD34^+^CD16/32^+^. Data are mean±SD *p<0.05, **p<0.01, ***p<0.00

Table 3. The percentage of bromodeoxyuridine in each population of the bone marrow from wild type, JAK2V617F/Vav-Cre, and JAK2V617F/Vav-Cre/Trp53^-/-^ mice.

| BM BrdU% | GMP | MEP | CMP | MPP | ST-HSC | LT-HSC |
| --- | --- | --- | --- | --- | --- | --- |
| C57Bl/6 | 27.8±7.93 | 43.55±17.04 | 25.28±5.34 | 12.13±5.69 | 6.7±2.21 | 2.33±1.24 |
| JAK2V617F/Vav-Cre | 32.07±5 | 48.57±14.86 | 22.39±11.99 | 18.5±10.36 | 9.27±2.85 | 13.2±1.44*** |
| JAK2V617F/VavCre/Trp53^-/-^ | 25.57±15.11 | 50.6±18.54 | 22.33±12.68 | 19.73±13.54 | 17±9.55 | 15.15±2.33*** |

GMP, Lin^+^sca^+^c-kit^+^CD34^+^CD16/32^+^; MEP, Lin^+^sca^+^c-kit^+^CD34^-^CD16/32^-^; CMP, Lin^+^sca^+^c-kit^+^CD34^+^CD16/32^-^; MPP, Lin^-^sca^+^c-kit^+^CD150^-^CD48^+^; ST-HSC, Lin^-^sca^+^c-kit^+^CD150^+^CD48^-^; and LT-HSC, Lin^-^sca^+^c-kit^+^CD150^-^CD48^-^. Data are mean±SD *p<0.05, **p<0.01, ***p<0.00

Table 4. The number of p53-dependent and -independent JAK2V617F-specific genes

|  | logFC JAK2 vs WT＞1.2  JAK2-specific | logFC JAK2p53-/- vs JAK2＜1.2  JAK2-specific p53-independent | logFC JAK2p53^-/-^ vs JAK2＞1.2  JAK2-specific p53-dependent |
| --- | --- | --- | --- |
| LT HSC | 3,496 | 1,363 (39%) | 2,133 (61%) |
| ST HSC | 3,995 | 1,282 (32.1%) | 2,713 (67.9%) |
| MPP | 7,417 | 5,315 (71.7%) | 2,102 (28.3%) |
| CMP | 4,276 | 1,447 (33.8%) | 2,829 (66.2%) |
| MEP | 4,229 | 2,113 (50%) | 2,116 (50%) |
| GMP | 3,549 | 961 (27%) | 2,588 (73%) |

LT-HSC, Lin^-^sca^+^c-kit^+^CD150^-^CD48^-^; ST-HSC, Lin^-^sca^+^c-kit^+^CD150^+^CD48^-^; MPP, Lin^-^sca^+^c-kit^+^CD150^-^CD48^+^; CMP, Lin^+^sca^+^c-kit^+^CD34^+^CD16/32^-^; MEP, Lin^+^sca^+^c-kit^+^CD34^-^CD16/32^-^; and GMP, Lin^+^sca^+^c-kit^+^CD34^+^CD16/32^+^

**Supplementary figures**

**
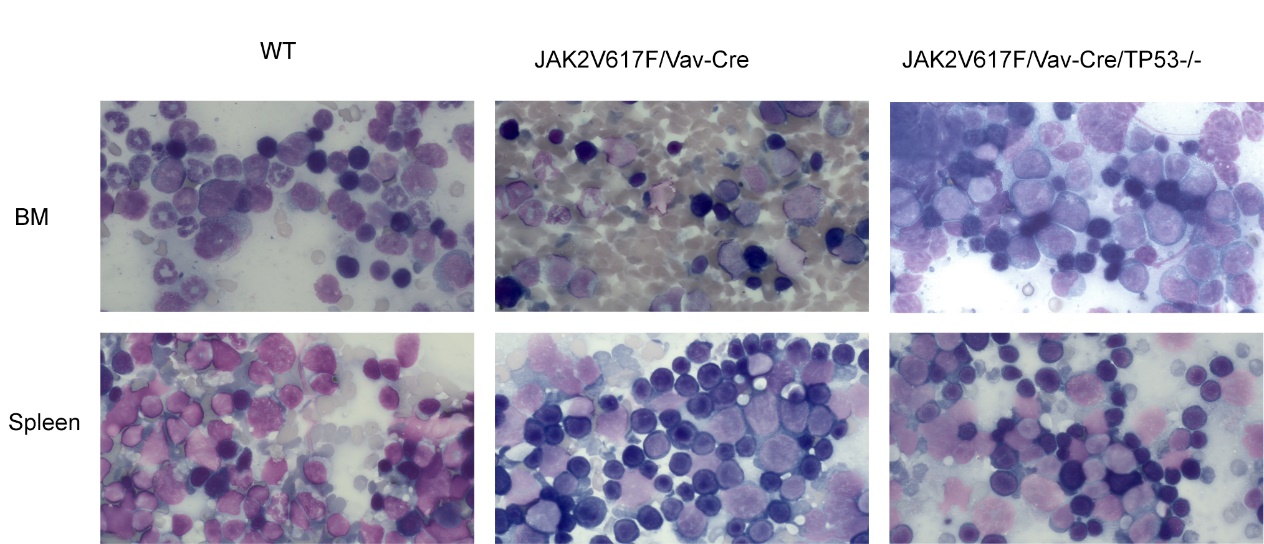
**

**Figure S1:** BM and spleen smears of WT, JAK2V617F/Vav-cre, and JAK2V617F/Vav-Cre/Trp53^-/-^. Images were obtained using a microscope with an Olympus camera.

**
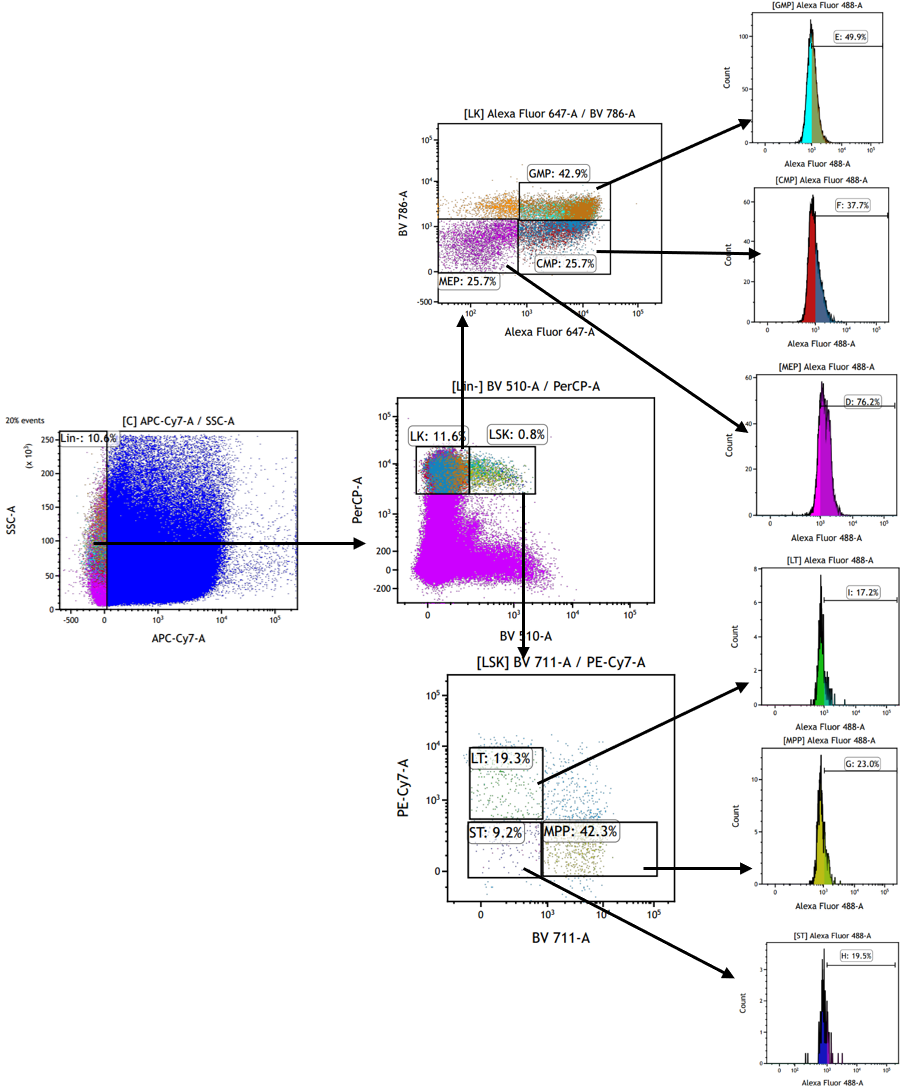
**

**Figure S2:** The gate strategy of flow cytometry analysis

**
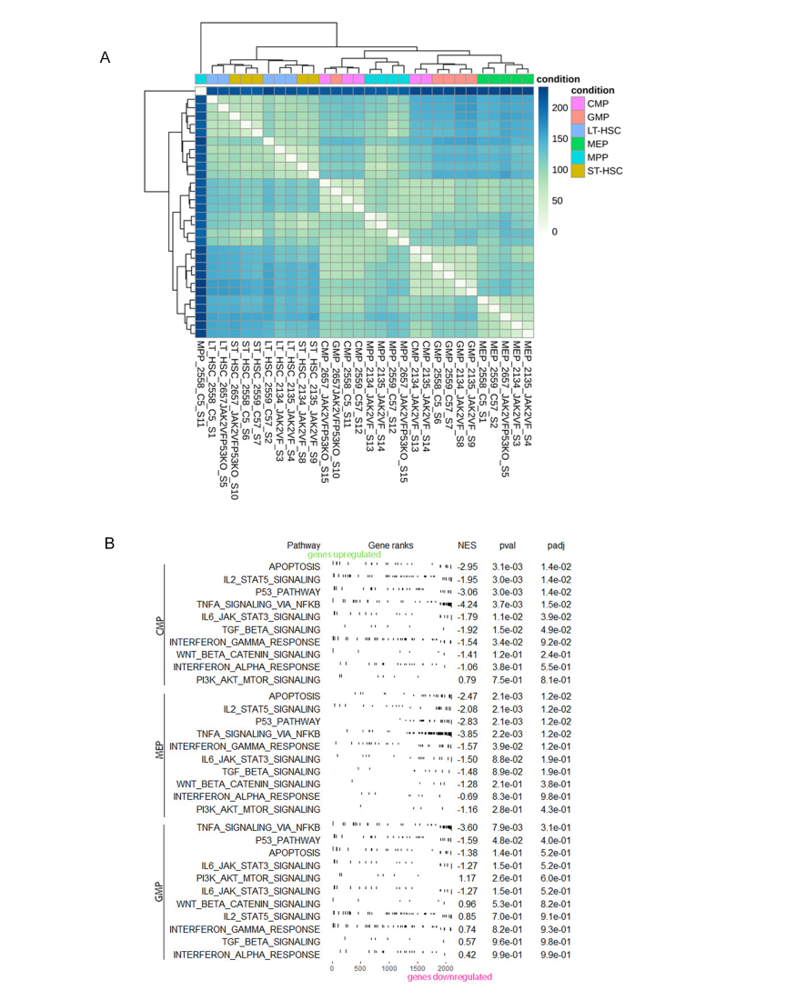
**

**Figure S3:** A, unsupervised analysis. B, GSEA analysis for Trp53 dependence in progenitor populations (CMP, GMP, and MEP).

**
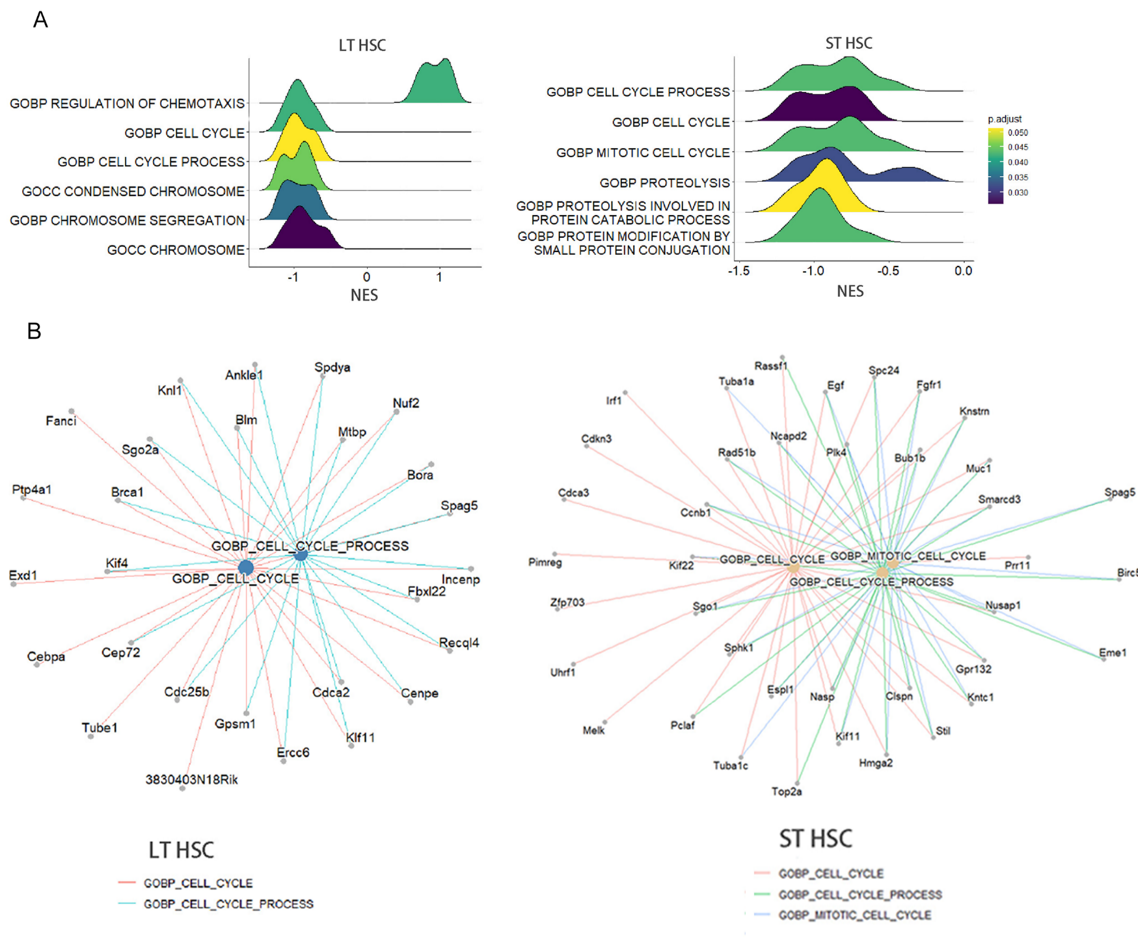
**

**Figure S4:** (A) GSEA analysis for p53 independence in LT-HSC and ST-HSC cells. (B) Network analysis of *Trp53*-independent genes in cell cycle pathway in LT-HSC and ST-HSC cells.
